# Assessment of safety and tolerability of remogliflozin etabonate (GSK189075) when administered with total daily dose of 2000 mg of metformin

**DOI:** 10.1186/s40360-021-00502-0

**Published:** 2021-06-13

**Authors:** Robert Dobbins, Elizabeth K. Hussey, Robin O’Connor-Semmes, Susan Andrews, Wenli Tao, William O. Wilkison, Bentley Cheatham, Katare Sagar, Barkate Hanmant

**Affiliations:** 1 Indivior, Inc., Durham, NC USA; 2 Nuventra, Inc., Durham, NC USA; 3 Parexel International, Durham, NC USA; 4 GlaxoSmithKline, Collegeville, PA USA; 5 Metavant Sciences, Durham, NC USA; 6 Avolynt, Inc., RTP, 3920 South Alston Avenue, Durham, NC 27713 USA; 7 Glenmark Pharmaceuticals Ltd, Mumbai, India

**Keywords:** Lactic acidosis, Metformin, Pharmacokinetics, Pharmacodynamics, Remogliflozin etabonate, Remogliflozin, Safety, SGLT2 inhibitor, Sodium-glucose transporters (SGLTs), Type 2 diabetes mellitus

## Abstract

**Background:**

Patients with type 2 diabetes mellitus (T2DM) are characterized by an elevated glycemic index and are at a higher risk for complications such as cardiovascular disease, nephropathy, retinopathy and peripheral neuropathy. Normalization of glycemic index can be achieved by dosing combinations of metformin with other anti-diabetic drugs. The present study (Clintrials number NCT00519480) was conducted to evaluate the safety, tolerability, pharmacokinetics and pharmacodynamics of remogliflozinetabonate, an SGLT2 inhibitor, withdoses (500 mg and 750 mg BID) greater than the commercial dose (100 mg BID)in combination with metformin with minimum daily dose of 2000 mg given in two divided doses.

**Methods:**

This was a randomized, double-blinded, repeat dose study in 50 subjects with T2DM. The study was conducted in three phases; run-in, randomization, and treatment. All subjects were on a stable metformin dosing regimen. Cohort 1 subjects were randomly allocated to receive either remogliflozin etabonate 500 mg BID or placebo BID (2:1) in addition to metformin. Cohort 2 subjects were administered with either remogliflozin etabonate 750 mg BID or placebo BID (2:1) in addition to metformin for 13 days. All the subjects were assessed for safety (adverse events, lactic acid levels, vital signs, electrocardiogram [ECG]), pharmacokinetic evaluation, and pharmacodynamics (Oral Glucose Tolerance Testing) parameters.

**Results:**

Co-administration of remogliflozin etabonate and metformin was well tolerated in all subjects during the observation period. There were no severe or serious adverse events (SAEs) and no increase in lactic acid concentration was reported during the study. The statistical results showed that concomitant administration of remogliflozin etabonate, either 500 mg or 750 mg BID, with metformin had no effect on the pharmacokinetics of metformin. The accumulation ratios, Day 13 vs. Day 1, for AUC values of remogliflozin etabonate and its metabolites were all very close to 1, indicating no accumulation in plasma concentrations of remogliflozin etabonate and its metabolites. Mean glucose values from baseline and glucose and insulin values following oral glucose tolerance test (OGTT) were decreased in all treatment groups.

**Conclusion:**

Co-administration of doses of remogliflozin etabonate (500 mg BID or 750 mg BID) greater than the commercial dose (100 mg BID) with metformin (2000 mg BID) was shown to be safe and effective during the observation period.

**Trial registration:**

ClinicalTrials.gov, NCT00519480. Registered:22 August 2007.

## Research in context

- **What is already known about this subject?**
  ➢ Patients with type 2 diabetes mellitus (T2DM) show an elevated glycemic index and are at a higher risk for serious complications, Metformin is a well known antidiabetic drug, Lower dose of Remogliflozin Etabonate in combination with metformin is safe and effective
- **What is the key question?**
  ➢ To evaluate whether higher doses Remogliflozin Etabonate in combination with metformin is safe and effective.
- **What are the new findings?**
  ➢ The co-administration of Remogliflozin Etabonate (500 or 750 mg BID) and Metformin (> 2000 mg/day) was found to be safe and tolerable in T2DM patients, with no metabolic disturbances observed.
  ➢ Body weight was reduced in all treatment groups; however, ∼1 kg more was lost in the RE groups compared to metformin alone.
  ➢ Observed reduction in fasting glucose and OGTT levels suggest potential improvement in glycemia with RE in patients already on high-dose of metformin
- **How might this impact on clinical practice in the foreseeable future?**
  ➢ As metformin is widely used antidiabetic agent, RE will be used in combination with metformin in treating subjects with T2DM. This study demonstrates safety and tolerability, and to some extent efficacy, in T2DM when RE and metformin is used for T2DM in clinical practice.

## Background

Type 2 diabetes mellitus (T2DM) is a chronic, metabolic disease with symptomatic hyperglycemia and is associated with a higher risk for complications such as cardiovascular disease, nephropathy, retinopathy and peripheral neuropathy [1]. As per the International Diabetes Federation, 425 million people have diabetes and this number is expected to rise to 629 million by 2045 [2]. Diabetic complications can be managed by keeping blood glucose levels within normal ranges. Treatment guidelines from the American Diabetes Association and European Association for the Study of Diabetes recommend metformin, along with diet and exercise, as a first line therapy followed by adding second-line agents to these patients with insufficient control of hyperglycemia [3]. However, despite the use of multiple medications to control patients’ blood glucose levels, two thirds of patients with T2DM remain unable to reach their HbA1c targets [4]. Therefore, development of new anti-diabetic drugs is needed but the combination or compatibility of metformin with other drugs becomes a serious challenge in developing new anti-diabetic therapies.

Metformin is an oral anti-hyperglycemic drug which decreases blood glucose levels by decreasing hepatic glucose production (gluconeogenesis), decreasing the intestinal absorption of glucose, and increasing insulin sensitivity by increasing peripheral glucose uptake and utilization [5]. Lactic acidosis is a serious adverse event (AE) associated with metformin administration and has been labeled as such as a warning by the FDA [6, 7]. Hyperglycemia can also be controlled by increasing renal glucose excretion via inhibition of sodium-glucose transporters (SGLTs) [8]. SGLT1 and SGLT2 are found in the kidney and are responsible for the majority of glucose reabsorption (10 and 90%, respectively). Vallon et al. reported that fractional renal reabsorption of glucose is reduced in mice lacking the SGLT2 gene as compared to wild-type mice [9]. Previously published studies with diabetic patients revealed SGLT2 inhibition lowers plasma glucose levels via urinary glucose excretion [10].

Remogliflozin Etabonate (RE) is a novel and potent inhibitor of SGLT2, and studies have reported a safe and effective response of RE in lowering the plasma glucose concentration in patients suffering with T2DM [11, 12]. In vitro analyses have also reported a low clinical drug interaction risk for remogliflozin etabonate because of availability of multiple biotransformation pathways [13].

In a previously published study, concomitant administration of RE (500 mg BID) and metformin (500 mg BID) for 3 days was well tolerated. Remogliflozin etabonate had no effect on the steady-state plasma profiles of metformin, and metformin had no effect on the pharmacokinetics of RE or its metabolites [14]. The present study was conducted to evaluate the safety, pharmacokinetics and pharmacodynamic analysis of concomitant administration of metformin (≥2000 mg / day) and RE (500 mg BID, 750 mg BID) over a longer duration (13 days) and in a larger number of (50) subjects with T2DM.

## Methods

### Study design

This was a randomized, double-blinded, repeat dose parallel group study. Subjects currently taking metformin for the treatment of T2DM and fulfilling the inclusion criteria were recruited. The study protocol was approved by the institutional review board (IRB) and the study was conducted in accordance with the major ethical principles specified in the Declaration of Helsinki and good clinical practice (GCP) guidelines. Written informed consent was obtained from each subject before any study related procedure was performed. This was a multicenter study conducted at a total of four centers: two centers in the USA, one center in Germany, and one center in Argentina. The study protocol, any amendments, the informed consent, and other information were reviewed and approved by relevant ethics committee or review board at each study center.

**Fig. 1:**
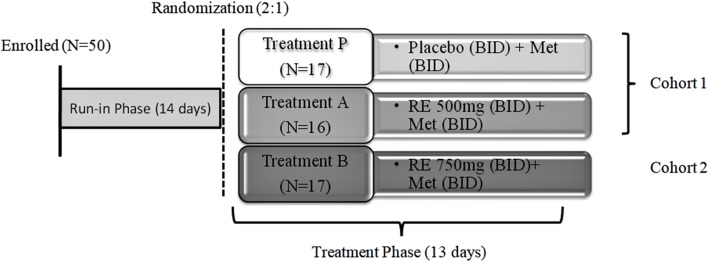
Summary Schematic of Study Design. Dosing of Cohort 2 commenced after at least 18 subjects in Cohort 1 had completed dosing and preliminary safety information had been reviewed. Two subjects were withdrawn; one for mild supraventricular tachycardia and one lost to follow-up. BID = twice daily; Met = metformin; RE = Remogliflozin etabonate

The study was comprised of three phases; run-in, randomization, and treatment. In the run-in phase, subjects who were taking < 2000 mg of metformin were titrated up over a 14-day period to a minimum daily dose of 2000 mg immediate release metformin. If subjects were taking metformin once daily (QD) or three times daily (TID), then they were titrated to BID for the duration of the study. Following the run-in phase, treatment phase of 13 days included in-house assessment in clinical research unit from day − 2 to day 4 and day 12 to day 14 and outpatient basis assessment from Day 5 to Day 11 with follow-up visit from Day 21–24. In treatment phase the patients were considered in two cohorts. Cohort 1 subject were randomly allocated to receive either RE 500 mg BID or placebo BID (2:1) in addition to metformin. Cohort 2 subjects received either RE 750 mg BID or placebo BID (2:1) in addition to metformin. Cohort 2 dosing commenced after at least 18 subjects in Cohort 1 had completed dosing and preliminary safety information had been reviewed (Fig. 1). The decision to begin Cohort 2 was based upon blinded review of AE reports, vital signs, ECG findings and clinical laboratory results by the site physicians and medical monitor.

### Treatment assignment

Treatment assignments were in accordance with the randomization schedule stratified by cohort with fixed block size. Upon successful completion of the metformin titration period, each subject is randomly assigned to one of the two cohorts with either one of the two RE dose regimens or placebo in a ratio of 2:1. A central randomization was applicable for all sites, and performed using a GSK proprietary IWRS randomization system.

### Sample size

The sample size planned for this study arose primarily from practical feasibility. There was no formal calculation of power or sample size. However, considering mean difference of plasma lactate level between treatment and placebo as one of evaluable end-points, a sample size estimation using the plasma lactate levels had been determined. A sample size of 12 subjects or 16 subjects with evaluable data completed for each active arm and placebo arm was estimated. This was based on larger inter-subject standard deviation of lactate level of 0.45 mmol/L in metformin alone group observed in previous report of 3-day metformin-RE drug-drug interaction study [15].

### Inclusion and exclusion criteria

Men and women (with adequate birth control, if of childbearing potential) aged 30–64 years (both inclusive) having a body mass index (BMI) of 22–40 kg/m^2^ with a documented T2DM diagnosis (diagnosed not less than 3 months; HbA1c range from 7 to< 10% and fasting plasma glucose (FPG) < 280 mg/dL at screening, fasting C-peptide lower limit of the reference range for an approved local laboratory assay) who were not adequately controlled by monotherapy with metformin were included in the study. Only those subjects were included who were willing and medically able to titrate their metformin dose to at least 2000 mg/day for 2 weeks, if on a lesser dose and have FPG > 126 mg/dL near the end of the run-in period.

Subjects with a prior history of autoimmune type 1 diabetes mellitus, diabetic ketoacidosis or lactic acidosis, gastrointestinal, hepatic or renal diseases were excluded from the study. Subjects requiring insulin therapy and any other anti-diabetic drug were also excluded from the study.

### Study objectives

The primary objective of the study was to access the safety and tolerability of RE (500 mg and 750 mg twice daily) along with metformin at doses 2000 mg daily in subjects with T2DM in terms of incident adverse events including hypoglycemia, plasma lactic acid levels and lactate levels and clinically relevant changes in vital signs and laboratory assessments.

The secondary objectives included the assessment of the effect of RE on steady state plasma concentrations of metformin, pharmacokinetics of RE and key metabolites while on metformin treatment and the response to an oral glucose tolerance test (OGTT) when RE was added to metformin versus metformin alone. The study assessment therefore included safety assessments (adverse events, hypoglycemia, plasma lactic acid level and lactic acidosis events, vital signs, ambulatory blood pressure monitoring, ECG, clinical laboratory values, fluid balance and body weight) as well as pharmacokinetic sampling, and plasma glucose, plasma insulin levels after OGTT.

### Adverse event monitoring

The adverse events monitoring included recording of any adverse event (AE) and serious adverse event (SAE) reported or observed during the entire study and follow-up period. All the subjects were counseled to inform study staff of any AEs that occurred during the study. Any abnormal laboratory findings (e.g., clinical chemistry, hematology, and urinalysis) or other abnormal assessments (e.g., ECGs, vital signs, physical examination) that were judged by the investigator as clinically significant were recorded as AEs or SAE.

### Clinical laboratory evaluations

For all measures, blood sample was obtained prior to having breakfast and were collected on baseline (Day-1), mornings (prior to first dose and breakfast) of Day 1, 2, 3, and 4 (inpatient) and Days 6, 8, and 10 (outpatient) and Days 13 and 14 (inpatient). Free fatty acids (FFAs) were also measured on Day-1 and 13. Samples were kept at 4 °C and were analyzed immediately.

### Surveillance for lactic acidosis

The samples for acidosis analysis were collected at screening (following an overnight fast and prior to morning dose), once during the run-in phase, at baseline (Day- 1), mornings (prior to first dose and breakfast) of Days 1, 2, 3, and 4 (inpatient) and Days 6, 8, and 10 (outpatient) and Days 13 and 14 (inpatient). Subjects were not allowed to do rigorous exercise or physical labor during the treatment phase of the study and instructed to limit exercise 12 h before sampling.

### Electrocardiogram

A 12-lead ECG was performed at the Screening Visit, Day- 1, pre-dose Days 4, 6, 8, 10, and 13; and at the follow-up Visit.

### Fluid balance assessments

The amount of all liquids consumed and urine volume were collected beginning the morning of Day 1 (prior to metformin dose) and the morning of Day 13 (prior to metformin and RE dosing), and continued until the subject was discharged from the clinical research facility after completing the inpatient portions of the study.

### Body weight

Body weight measurement was performed at screening and during inpatient Days- 1, 2, 4, 8, 13, and 14.

### Ambulatory blood pressure monitoring

The ambulatory blood pressure was monitored for 24 during inpatient days (beginning Day 1 at 08.00, Day 2 at 08.00 and Day 13 at 08.00). The unit was programmed to take blood pressure readings every 10 min from 08.00 to 10.00, every 20 min from 10.00 to 22.00, and every 30 min from 22.00 to 08.00 the following morning.

### Pharmacokinetic analysis

Serial blood samples (2 ml) were collected on Day 1 and Day 13 at different time-points for the determination of remogliflozin etabonate, remogliflozin (GSK189074), GSK279782 and metformin concentrations in plasma; pre-dose (within 10 min prior to next dose), 15, 30, 45 min and 1, 1.5, 2, 2.5, 3, 4, 6, 8, 10 and 12 h (before second dose of the day). PK analysis was conducted by INDAPharma, LLC (Chapel Hill, NC, USA), under the direction of Clinical Pharmacokinetics/Modelling and Simulation, GSK using the non-compartmental analysis Model 200 (for extra vascular administration) of WinNonlin Professional Edition version 4.1 (Pharsight Corporation, Mountain View, CA). The drug assay techniques utilized for each of the drug and metabolites has been reported prior [14].

### Pharmacodynamic analysis

Oral glucose tolerance testing was performed on Day 1 and Day 13 using a 75 g oral glucose solution. Blood samples for the measurement of insulin and glucose were taken immediately before drug dosing (within 5 min of drug dosing), and at 30 min, 1, 1.5, 2, 3, 4, and 6 h following drug dosing.

### Statistical analysis

Descriptive statistics were used to summarize baseline demographics, safety data, and pharmacokinetic parameters. Geometric mean and % CV_b_ (between-subject coefficient of variation) were calculated for all pharmacokinetic parameters except t_max_. Comparisons of placebo with each active dose of RE were assessed in terms of change from baseline in fasting plasma lactate acid using repeated measures analysis of covariance model. The model included terms for baseline measurement, visit, treatment and visit-by-treatment interaction. The same model was applied to change from baseline in weighted means of day-time, night-time and 0–24 h of blood pressures. Within group treatment comparison was performed using mixed model on log transformed C_max_ and AUC _(0-last)_.

## Results

A total of 50 subjects were enrolled in the study. Two subjects were withdrawn from the study; one due to an AE (supraventricular tachycardia) and one was lost to follow-up. Thus, 48 subjects (men [44%], women [56%]) completed the study as planned and were included in the analyses. The mean age of the included patients was 54 years (range 40 to 60 years) and the mean BMI was 30 kg/m^2^ (range23–40 kg/m^2^). The mean duration of exposure to remogliflozin etabonate was 12.4 days during RE 500 mg + metformin, 12.7 days during RE 750 mg + metformin, and 13 days during placebo treatment.

### Safety and tolerability

The occurrence of adverse events by the treatment groups have been enlisted in Table 1. All AEs reported during the study were mild in intensity and resolved spontaneously. One subject was withdrawn from the study on Day 3, due to an AE of supraventricular tachycardia, which was not an SAE. The most commonly reported AEs were diarrhea (10%; *N* = 5/50), nausea (6%; *N* = 3/50) thirst, vomiting and dizziness (4%; *N* = 2/50). After treatment, urinary tract infection and dyslipidemia were reported in only 1 subject from each placebo and RE 500mg + metformin groups, respectively. The group administered with RE 750 mg + metformin reported no post-therapy AEs. Two subjects had hypoglycemic laboratory events during the study, one each in placebo + metformin group (mild, resolved without intervention) and RE 750 mg + metformin group (moderate, resolved with sweets). No other clinical laboratory values were reported as AEs in the run-in phase, or during the treatment Phase. No significant changes or trends in vital signs or ECGs were observed during the treatment period.

**Table 1:** Analysis of All Drug-Related and Post-Therapy Adverse Events

Adverse Event	Placebo + Metformin (*N* = 17)	RE 500 mg + Metformin (*N* = 16)	RE 750 mg + Metformin (*N* = 17)
All Adverse Event (AE) N(%)			
Any AE	3 (18%)	6 (38%)	4 (24%)
Diarrhoea	1 (6%)	3^a^ (19%)	1 (6%)
Nausea	1^a^ (6%)	1 (6%)	1 (6%)
Thirst	1 (6%)	0	1^a^ (6%)
Vomiting	0	1^a^ (6%)	1 (6%)
Dizziness	0	1 (6%)	1 (6%)
Supraventricular tachycardia	0	1^a^ (6%)	0
Urinary tract infection	1^b^ (6%)		0
Dyslipidemia	0	1^b^ (6%)	0

*AE* Adverse event, *RE* Remogliflozinetabonate ^a^ determined to be drug related adverse event, one of 3 events of diarrhoea with RE 500 mg + Met determined to be drug related, ^b^ post therapy adverse events occurred after last dose

**Fig. 2:**
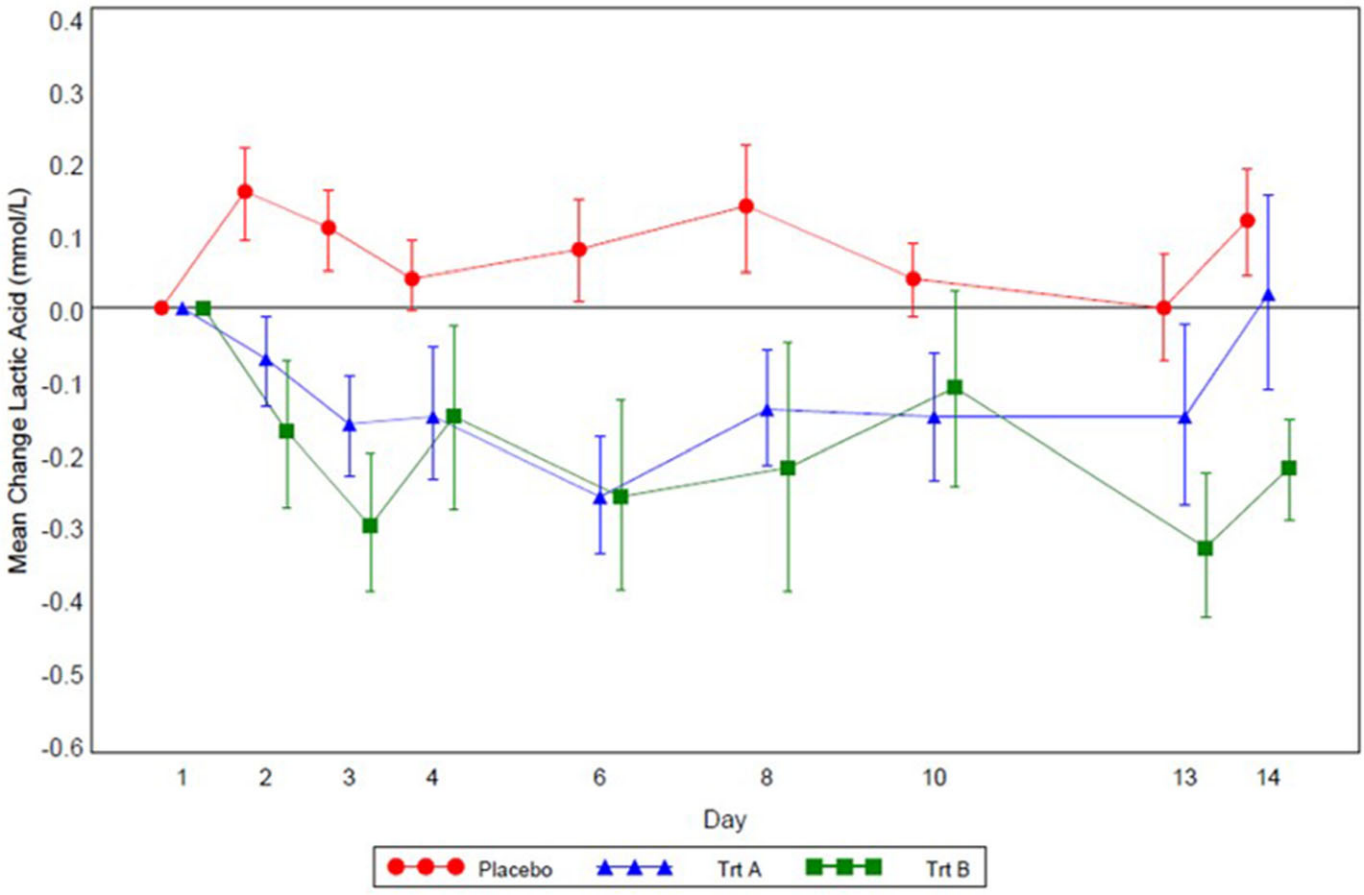
Plot of Mean (± standard error) Change from Baseline in Fasting Plasma Lactic Acid Concentration (mmol/L). Trt A, Remogliflozin Etabonate 500 mg + metformin; Trt B, Remogliflozin Etabonate 750 mg + metformin

Mean plasma fasting lactic acid decreased over time during dosing from Day 2 to Day 13 for subjects in Cohort 1 and 2 and appeared to increase slightly from Day 2 to Day 13 in the placebo group (Fig. 2). The mean maximum on-therapy fasting lactic acid values were not increased in the RE-containing treatments compared to placebo treatment (Table 2).

**Table 2:** Summary of Fasting Plasma Lactic Acid Concentration

Lactic acid (mmol/L)	Placebo + Metformin (*N* = 17)	RE 500 mg + Metformin (*N* = 16)^a^	RE 750 mg + Metformin (*N* = 17)^b^
A. Summary of Maximum On-Therapy Absolute concentrations and Changefrom Baseline; Mean (SD)			
Absolute Values	1.554 (0.5094)	1.220 (0.2684)	1.508 (0.4389)
Change From Baseline	0.460 (0.2289)	0.234 (0.3717)	0.249 (0.5934)
B. Summary of Repeated measures analysis ofcovariance of change from baseline (vs. placebo) on selected days			
Study Day 2			
Mean (SD)	0.16 (0.266)	−0.07 (0.248)	−0.17 (0.419)
Difference (95% CI)		− 0.284 (− 0.485, − 0.083)	−0.246(− 0.445, − 0.047)
*p-*value		0.0066	0.0164
Study Day 6			
Mean (SD)	0.08 (0.288)	−0.26 (0.310)	−0.26 (0.538)
Difference (95% CI)		−0.380 (− 0.603, − 0.157)	−0.254(− 0.472, − 0.036)
*p-*value		0.0013	0.0232
Study Day 13			
Mean (SD)	0.00 (0.304)	−0.15 (0.485)	−0.33 (0.398)
Difference (95% CI)		−0.191 (− 0.414, 0.031)	−0.255 (− 0.475, − 0.034)
*p*-value		0.0905	0.0244

*CI* Confidence interval, *RE* Remogliflozin Etabonate, *SD* Standard deviation; ^a^: n = 15 for Day 6 & 13; ^b^: n = 16 for Day 13

The treatment vs placebo difference is based on ANCOVA: Change = Baseline + Treatment + Visit + Treatment-by-Visit

Statistically significant differences (decreases) in change from baseline fasting plasma lactic acid were observed between the two treatment groups versus placebo on most Study Days. In terms of events, five subjects had a lactic acid greater than the upper limits of normal (ULN) at anytime during the study; in four subjects the elevations occurred while receiving metformin alone and in one subject receiving RE. None of the elevations were sustained and all were minimally above the ULN. No subject was withdrawn for increased lactic acid or any other metabolic abnormality.

Ambulatory blood pressure monitoring measured decreases from baseline in systolic and diastolic readings at nighttime (midnight to 6 am) on Day 13 were significantly different for both RE regimens compared to placebo (metformin alone). The systolic blood pressure decreased significantly by 8.4 mmHg for the RE 500 mg and 7.1 mmHg for the RE 750 mg in Night time. Likewise, diastolic blood pressure decreased significantly by 6.5 mmHg for the RE 500 mg and 4.8 mmHg for the RE 750 mg in night time. However, reductions observed over the daytime (10 am to 8 pm) and the entire 24-h periods were not statistically significant (Table 3).

**Table 3:** Summary of AmbulatoryBlood pressure monitoring

Time	Reading (Adjusted Mean Change from Baseline inmmHg; *p*-value)	RE 500 mg + Metformin(N = 14)	RE 750 mg + Metformin(*N* = 14)
24 h	**Systolic**	−2.8; *p* = 0.303	−4.0; *p* = 0.131
	**Diastolic**	−2.0; *p* = 0.256	−3.3; *p* = 0.062
Daytime	**Systolic**	−3.4; *p* = 0.291	−2.4; *p* = 0.441
	**Diastolic**	−1.8; *p* = 0.461	−1.3; *p* = 0.586
Nighttime	**Systolic**	−8.4; *p* = 0.020	−7.1; *p* = 0.047
	**Diastolic**	−6.5; *p* = 0.006	−4.8; *p* = 0.043

*BP* Blood pressure, *RE* Remogliflozin Etabonate

The changes were ANCOVA adjusted mean change from baseline to Day 13 vs. Placebo

### Pharmacokinetics

The metformin mean AUC _(0-tau)_ [13,895 vs 14,693 (hr.ng/mL)] and C_max_ [2162 vs 2166 (ng/mL)] values were comparable between Day-1 (metformin alone) and Day 13 (RE 500 mg + metformin group); mean AUC _(0-tau)_ ratio (90% CI) was 1.03 (0.97, 1.09) (Table 4). Median t_max_ values were also equivalent [3.0 vs 2.5 h]. The metformin mean AUC _(0-tau)_ [11,319 vs 11,392 (hr.ng/mL)] and C_max_ [1758 vs 1695 (ng/mL)] values were comparable between Day-1 (metformin alone) and Day 13 (RE 750 mg + metformin group). The mean AUC _(0-tau)_ ratio (90% CI) was 1.03 (0.97, 1.09) and 1.01 (0.88, 1.16) for the RE 500 mg + metformin and RE 750 mg + metformin groups, respectively (Table 5).

**Table 4:** Summary of Pharmacokinetic Parameters of Metformin

Treatment	Day- 1 Metformin alone			Day 13 Metformin + RE		
	AUC (0-tau) (hr.ng/mL)	C_**max**_ (ng/mL)	t_**max**_ (hr)	AUC (0-tau) (hr.ng/mL)	C_**max**_ (ng/mL)	t_**max**_ (hr)
Metformin +	12,305	1967	2.0	13,955	2041	2.5
Placebo	(20)	(22)	(0.5–4.0)	(24)	(24)	(0.75–8.0)
Metformin +	13,895	2162	3.0	14,693	2166	2.5
RE 500 mg	(24)	(24)	(0.5–4.0)	(25)	(28)	(0.75–4.0)
Metformin +	11,319	1758	2.5	11,392	1695	2.0
RE 750 mg +	(33)	(26)	(0.5–4.0)	(37)	(34)	(0.5–4.1)

*RE* Remogliflozin Etabonate

Values are geometric mean (%CVb) for each parameter, except for t_max_ which are median (range)

**Table 5:** Statistical Analysis of accumulation ratio basis PK parameters of Metformin

PK Parameter	RE 500 mg + Metformin (Day 13/Day-1)	RE 750 mg + Metformin (Day 13/Day-1)
**AUC**_**(0-tau)**_ **(hr.ng/ml)**	1.03 (0.97, 1.09)	1.01 (0.88–1.16)
**C**_**max**_ **(ng/ml)**	0.98 (0.9, 1.08)	0.96 (0.84–1.10)

*PK = RE* Remogliflozin Etabonate

Values are point estimate (90% confidence interval) of the geometric least-square mean ratio, Day 13 versus Day- 1

In the RE 500 mg group, remogliflozin, the active form of the prodrug, was formed with peak concentrations ranging from 0.5 to about 6.0 h. The mean AUC _(0-tau)_ [4894 vs 4928 (hr.ng/mL)] and C_max_ [1999 vs 1494 (ng/mL)] values were comparable between Day 1 and Day 13 (Table 6). The mean AUC_(0-tau)_ accumulation ratio (90% CI) was 1.00 (0.91, 1.10) for RE 500 mg + metformin (Table 7).

**Table 6:** Summary of Pharmacokinetic Parameters of Remogliflozin Etabonate and its Metabolites

Analyte	Day 1 RE 500 mg BID(*N* = 16)			Day 13 RE 500 mg BID(*N* = 15)		
	**AUC**_**(0-tau)**_ **or AUC**_**(0-last)**_^**a**^ **(hr.ng/ml)**	**C**_**max**_ **(ng/ml)**	**t**_**max**_ **(hr)**	**AUC**_**(0-tau)**_ **or AUC**_**(0-last)**_^**a**^ **(hr.ng/ml)**	**C**_**max**_ **(ng/ml)**	**t**_**max**_ **(hr)**
RE	70.9 (45)	64.8 (55)	0.63 (0.25–3.0)	73.5 (47)	53.3 (94)	0.50 (0.25–4.0)
Remogliflozin	4894 (40)	1999 (60)	0.77 (0.50–6.0)	4928 (33)	1494 (39)	3.0 (0.5–4.0)
GSK279782 (active metabolite)	878 (44)	257 (51)	1.3 (0.75–6.0)	1053 (44)	251 (49)	4.0 (0.5–4.0)
Analyte	**Day 1** **RE 750 mg BID (*N*** **= 17)**			**Day 13** **RE 750 mg BID (*N*= 16)**		
RE	121 (39)	137 (74)	0.50 (0.25–2.5)	121 (41)	101 (74)	0.48 (0.22–2.6)
Remogliflozin	8515 (31)	4008 (46)	0.75 (0.50–2.5)	6996 (39)	2508 (65)	1.3 (0.50–4.0)
GSK279782 (active metabolite)	1600 (50)	528 (43)	1.0 (0.75–3.0)	1298 (42)	326 (49)	3.0 (0.6–4.1)

*BID* twice daily, *RE* Remogliflozinetabonate

Values are geometric mean (%CV_b_) for each parameter, except for t_max_ which are median (range)

^a^AUC_(0-tau)_ for remogliflozin and GSK279782; AUC_(0-last)_ for remogliflozin Etabonate

**Table 7:** Statistical Analysis of accumulation ratio basis PK Parameters of RemogliflozinEtabonate and Its Metabolites

PK Parameter	RE 500 mg + Metformin			RE 750 mg + Metformin		
	RE	Remogliflozin	GSK279782	RE	Remogliflozin	GSK279782
**AUC**_**(0-tau)**_ **or AUC**_**(0-last)**_^**a**^ **(hr.ng/ml)**	0.99 (0.857–1.15)	1.00 (0.907–1.10)	1.17 (1.03–1.34)	1.02 (0.861–1.21)	0.82 (0.755–0.899)	0.84 (0.760–0.937)

*RE* Remogliflozin Etabonate

Values are point estimate (90% confidence interval) of the geometric least-square mean ratio, Day 13 versus Day 1.

^a^AUC_(0-tau)_ for remogliflozin and GSK279782; AUC_(0-last)_ for RE

In the RE 750 mg group, the remogliflozin mean AUC_(0-tau)_ [8515 vs 6996 (hr.ng/mL)] and C_max_[4008 vs 2508 (ng/mL)] values were modestly different between Day 1 and Day 13 (Table 6). The mean AUC_(0-tau)_ accumulation ratio (90% CI) was 0.82 (0.755, 0.899) (Table 7).

### Pharmacodynamics

The mean plasma glucose values for RE 500 mg + metformin and RE 750 mg + metformin were decreased from 13 to 9 mmol/L and 11 to 8 mmol/L within 13 days, respectively. Similarly, the mean insulin values were also decreased in all the three treatment groups (Table 8).

**Table 8:** Evaluation of Pharmacodynamic Parameters

Parameter	Placebo + Metformin (N = 17)	RE 500 mg + Metformin ^b^	RE 750 mg + Metformin (*N* = 17)
Change from Baseline Weighted Mean Plasma Glucose and Insulin			
Glucose (mmol/L), Day 1			
Mean (SD)	12.65 (3.306)	13.03 (1.929)	11.49 (3.536)
Glucose (mmol/L), Day 13			
Mean (SD)	11.49 (3.536)	9.45 (1.502)	8.84 (1.656)
Glucose, Change from Baseline			
Mean (SD)	−0.10 (1.572)	−3.50 (2.012)	−2.77 (2.262)
Insulin (pmol/L), Day −1			
Mean (SD)	176.85(104.735)	190.96 (100.420)	191.52 (83.045)
Insulin (pmol/L), Day 13			
Mean (SD)	162.97(97.374)	142.15 (67.626)	171.57 (74.178)
Insulin, Change from Baseline			
Mean (SD)	−13.88(42.349)	−44.80 (52.746)	−18.95 (47.360)
Change from Baseline in Body Weight (kg)			
Day 2, Mean (SD)	−0.70 (0.679)	−1.03 (0.994)	−1.04 (1.210)
Day14, Mean (SD)	−1.02 (0.845)	−2.03 (1.235)	−1.84 (1.398)
Change in Fluid Balance (ml)			
Day 2, Mean (SD)	203.5 (999.83)	− 335.3 (678.37)	− 110.2 (1064.69)
Day13, Mean (SD)^a^	−42.0 (1262.56)	− 610.1 (1631.47)	− 416.9 (493.86)

*RE* Remogliflozin Etabonate, *SD* Standard deviation

^a^Analyzed subjects on Day 13 for RE 500 mg + Metformin group were 15, ^b^Analyzed subjects on day 13 for RE 750 mg + Metformin group were 16

Body weight decreased in all treatment groups including placebo; however, subjects who received RE lost 1 kg more weight, (− 2.03 kg for RE 500mg + metformin and − 1.84 kg for RE 750 mg + metformin) by Day 14 than those who received placebo (− 1.02 kg for placebo + metformin) (Table 8).

Total fluid intake, total urine volume, and fluid balance (intake – urine output) were also recorded during the study and these values were highly variable in all treatment groups.

## Discussion

For patients with T2DM, metformin-based combination therapy is recommended when monotherapy is not enough to achieve targeted glycemic control [3]. Multiple doses and combinations of anti-hyperglycemic agents with negligible drug-drug interactions are preferred. This study was conducted to access the potential for metabolic disturbances that can lead to severe AEs in combination therapy [16, 17]. In a previous study, the safety and effectiveness of a combination of RE (500 mg BID) with metformin (500 mg BID) in13 subjects with T2DM for a duration 3 days was reported with no safety signals [14]. The aim of the current study was to evaluate the safety, pharmacokinetics and pharmacodynamics of concomitant administration of higher doses of metformin (≥2000 mg total daily dose) with RE 500 mg BID and 750 mg BID over a longer duration (13 days) and in a larger number of (50) patients with T2DM.The current analysis too did not observe any safety signals on adverse events, hypoglycemia or laboratory or ECG assessments on concomitant administration of high dose ranges of RE and metformin ≥ 2000 mg daily.

One potentially life-threatening side effect of metformin is lactic acidosis, as metformin in therapy increases the fasting plasma lactate in patients with T2DM [14, 18]. The previously published study reported a decrease in plasma lactic acid concentration, during co-administration of RE with metformin, and no symptoms of lactic acidosis were observed [14]. In the current analysis, a decrease in fasting plasma lactic acid concentrations with the addition of RE to subjects stably dosed with metformin was observed. No subjects were withdrawn from this study for an elevated lactic acid or metabolic abnormality.

The inhibition of SGLT2 in the kidney with resulting increases in glycosuria a relatively new therapeutic modality for the treatment of subjects with T2DM. Remogliflozin etabonate is an orally available, novel, potent inhibitor of SGLT2, and has been shown in numerous clinical trials to be a safe and efficacious monotherapy for the treatment of T2DM [12, 19–21].

O’Connor-Semmes et al. have demonstrated the repeat dosing PK of RE showed only 10% increase in AUC and t_1/2_, indicating no accumulation of RE in plasma concentrations [20]. Similarly, Mikhail et al. opined that RE and its metabolites (remogliflozin and GSK279782) not only lowers the blood glucose levels but have also relatively short half-lives; thus they do not accumulate in plasma [10]. This study examined any possible changes in PK of RE with concomitant metformin and vice versa.

Data from several clinical studies are highly suggestive that SGLT2 inhibitors, as a class, appear to be safe and efficacious when combined with metformin to treat T2DM [22]. Consistent with these earlier observations [14], RE does not interfere with PK parameters of metformin; the mean AUC_(0-tau)_ and C_max_ values of metformin were found to be comparable between the three groups. Remogliflozin Etabonate was rapidly absorbed following concomitant oral administration with metformin and the active metabolite remogliflozin was also rapidly formed with peak concentration ranging from 0.5 to about 4 h. The accumulation ratios, Day 13 vs. Day 1, for AUC values of RE and its metabolites showed variable observations with the two dose levels tested. However, on background of PK of RE it is important to note that all ratios were all close to 1, indicating no accumulation in plasma concentrations of RE and its metabolites following multiple-dose administration with concomitant metformin and not indicative of clinically significant changes in plasma exposure. The PK parameters of RE and its metabolites following 750 mg BID dose with metformin were nearly dose proportional to those following the 500 mg BID dose of RE with metformin. This steady-state plasma profiles of RE and its metabolites observed in this study were comparable to the dose-normalized PK parameter values found previously in T2DM subjects without or with concomitant metformin. These data indirectly suggest that no effect of metformin on the PK profiles of RE and its metabolites, as direct within-study comparison was not possible as RE was only administered with metformin and not alone.

The observation of decrease in the AUC_(0-tau)_ accumulation ratio for remogliflozin in the 750 mg group would suggest a potential impact on the efficacy of RE. However, the pharmacodynamic assessments showed significant results of RE when concomitantly administered with metformin. Similarly, the Phase III study of RE [23] observed glycemic efficacy comparable to dapagliflozin when administered in T2DM patients on daily metformin therapy (> 1500 mg) over 24 weeks. As the efficacy and safety profile of RE is not seen to be affected and clinical doses of RE is lower (100 mg BID), the observed variations in accumulation ratio can be considered of low clinical relevance.

Results from the pharmacodynamic analysis revealed that mean fasting glucose values decreased in both RE treatment groups. Reductions in the weighted mean glucose and insulin following an OGTT were also observed, suggesting that the addition of RE may significantly improve glycemia in subjects already taking a higher dose of metformin [23].

Furthermore, a clear reduction in body weight in all the treatment groups was observed. However, the RE cohorts demonstrated a 1 kg loss more compared to metformin alone. Weight loss could have resulted from changes in both fluid and caloric balance.

Patients with T2DM often have hypertension, which the combination increases the risk for cardiovascular complications. Thus, apart from glycemic control, the modification of cardiovascular risk factors is also important in the management of T2DM. According to the European Society of Hypertension, extreme dipping in case of ambulatory BP can result in stroke [24]. Therefore, regulation of BP likely helps in lowering the risk for cardiovascular complications [25]. In the present analysis, ambulatory BP monitoring measured statistically significant decreases from baseline in systolic (8.4 and 7.1 mmHg) and diastolic (6.5 and 4.8 mmHg) readings at nighttime on Day 13 for both RE regimens compared to placebo (metformin alone).

The present study revealed that higher doses of RE than used commercially and metformin were generally well tolerated and severe AEs were not reported. The most commonly reported AEs were diarrhea, nausea, thirst, vomiting, and dizziness. This indicates that concomitant administration of RE, either 500 mg BID or 750 mg BID; with metformin 2000 mg (total daily dose) had no effect on the steady-state plasma profiles or pharmacokinetics of metformin.

Addition of RE to ongoing metformin therapy offers a potential for therapeutic benefit without a significant risk of lactic acidosis, hypoglycemia or other AEs. However, as the study was conducted in feasible sample size, the interpretations would need to be considered exploratory in nature and would require independent evaluation in studies planned with statistically relevant sample sizes.

## Conclusion

The present study concludes that concomitant administration of RE, either 500 mg or 750 mg BID, with metformin 2000 mg BID is safe and effective in patients with T2DM during the observation period. Remogliflozin Etabonate does not affect the PK profile of metformin and improves the plasma blood glucose levels by increasing the excretion of urine glucose.

## Data Availability

All the relevant data has been represented in the manuscript. Any additional data can be provided by corresponding author on reasonable request.
